# The Dielectrophoretic Alignment of Biphasic Metal Fillers for Thermal Interface Materials

**DOI:** 10.3390/polym15244653

**Published:** 2023-12-08

**Authors:** Yangwoo Lee, Kubra Akyildiz, Chanmi Kang, Ju-Hee So, Hyung-Jun Koo

**Affiliations:** 1 Department of Chemical & Biomolecular Engineering, Seoul National University of Science & Technology, Seoul 01811, Republic of Korea; diddnqkqh@gmail.com (Y.L.);; 2 Material & Component Convergence R&D Department, Korea Institute of Industrial Technology, Ansan 15588, Republic of Korea

**Keywords:** thermal interface materials, composites, dielectrophoretic chaining, liquid metal

## Abstract

Pad-type thermal interface materials (TIMs) with composite structures are required to exhibit high thermal conductivity while maintaining conformal contact with the heat sink, which is strongly influenced by the type and content of the thermally conductive filler. This study presents that biphasic metal particles can be effectively aligned using the dielectrophoretic chaining (DEP-C) mechanism, thereby enhancing the thermal conductivity of a pad-type TIM. A eutectic gallium–indium (EGaIn) alloy liquid metal and solid copper were used as the filler materials with two different phases. The biphasic metal particle mixture of EGaIn and Cu (EGaIn-Cu) were better aligned by DEP-C than when they presented individually because fusion between the two particles increased the effective size. As expected, the thermal conductivity of the TIM composites increased when DEP-C aligned the filler. Notably, TIMs with both EGaIn-Cu fillers showed the largest increase in thermal conductivity, of up to 64.6%, and the highest thermal conductivity values after DEP-C application compared to TIMs with only the EGaIn or Cu filler. Finally, the heat dissipation performance of the TIM composite on a lit light-emitting diode is shown, where the TIM with DEP-C-aligned fillers exhibits improved performance.

## 1. Introduction

Recently, emerging technologies, such as artificial intelligence, autonomous driving, and electric vehicles, require highly integrated circuits [1,2]. As the integration density of the circuit increases, the amount of heat generated drastically increases, which not only degrades the performance and life span of the device but also causes accidents, such as fires [3,4,5]. Generally, aluminum- or copper-based heat sinks are widely used for thermal management because these metals can effectively dissipate heat, owing to their high thermal conductivity (237 W/(m·K) and 398 W/(m·K) for aluminum and copper, respectively). However, heat sinks made of rigid metals exhibit poor thermal contact with heat sources due to their rigidity [6]. Thermal interface materials (TIMs) have been utilized as a form of paste, adhesive, or pad to resolve the thermal resistance between heat-producing components and heat sinks. Paste- and adhesive-type TIMs usually consist of a base material, such as silicone or hydrocarbon, and thermally conductive fillers. Usually, these types of TIMs are coated between the heat sink and the heat source and effectively fill in uneven gaps at the interface because of the rheological properties of the pastes and adhesives. However, liquid-type TIMs may be challenging to apply reliably and require additional fixing or curing processes. Conversely, pad-type TIMs are a soft solid form of film composed of a silicone or polymer matrix with embedded metal fillers. The pad-type TIMs are easy to assemble and disassemble and accommodate non-flat interfaces between heat sources and heat sinks [7].

The desire to develop pad-type TIMs with high thermal conductivity has attracted considerable research efforts on composites with different types of thermally conductive fillers, such as boron nitride [8,9,10], aluminum oxides [11], aluminum nitride [10,12], copper [13,14], graphite [15,16], graphene [17,18], and carbon nanotubes [13,18,19]. However, composites with these fillers exhibit substantially lower thermal conductivity compared to solid metals due to the polymer matrix. The silicone materials that are typically used as the polymer matrix for pad-type TIMs have a thermal conductivity of 0.16–0.20 W/(m·K) [20,21], which is two to three orders of magnitude lower than that of solid metals. However, as the filler content increases to improve the thermal conductivity of the composite, the TIM pads become stiffer, hindering conformal contact with the heat source or heat sink. Not only is there a trade-off between flexibility and thermal conductivity, but also the composite itself may not form if the concentration of the filler reaches a certain point. Therefore, developing a technique is crucial to achieve the high thermal conductivity of TIMs with a proper filler content.

Liquid metals that are in a liquid state at room temperature are promising candidates as filler materials for pad-type TIMs because they can be used in a high content with a minimal loss of composite softness [22]. Moreover, as liquid metals deform easily in response to external physical stresses, TIMs with liquid metal fillers can potentially be applied to flexible and stretchable devices [23,24]. In particular, the liquid metal eutectic gallium–indium (EGaIn) alloy is in the spotlight due to its advantages, such as a low melting point (15.5 °C), low viscosity, low vapor pressure, low toxicity, and good thermal conductivity (26.4 W/(m·K)) [25]. To explore these advantages, studies on liquid metal-based composites for TIMs are in progress, such as the surface modification of liquid metal particles to restrain the shape deformation and coalescence [6] or reshaping the liquid metal particles into nano/microchannels using external stresses to improve the anisotropic thermal conductivity [6,26]. EGaIn is also known to have good wettability with other metals and form an alloy, which can lead to better connectivity between the metal particles and improve the thermal conductance property [27,28,29,30].

**Figure 1:**
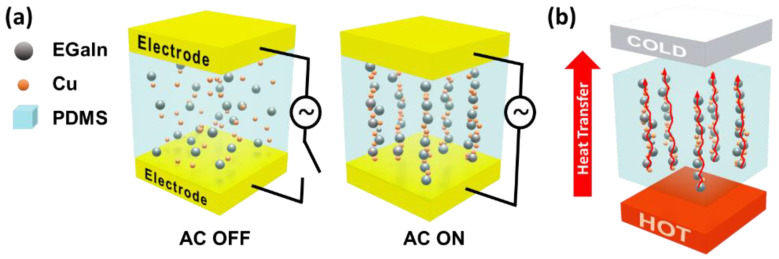
Dielectrophoretic alignment of EGaIn and Cu metal particles in the composites for pad-type TIMs; (**a**) dispersed EGaIn and Cu conductive fillers in the PDMS matrix with and without alternating current (AC) field; (**b**) directional heat transfer induced by the linear alignment of the biphasic metal fillers.

This study presents TIM composites composed of two metals with different phases, namely liquid EGaIn and solid copper (Cu) microparticles, dispersed in a polydimethylsiloxane (PDMS) matrix, as shown in Figure 1a. Herein, TIMs are defined as EGaIn-Cu/PDMS TIMs. Furthermore, an electric field is applied to induce the directional thermal conductivity in TIMs by aligning the “biphasic” metal fillers (Figure 1b) [26,31]. Particles under the electric field are linearly aligned and connected along the field direction, which is called dielectrophoretic chaining (DEP-C). It is hypothesized that the biphasic metal fillers aligned by DEP-C are connected and fused, thereby enhancing the directional heat transfer. Cu particles were chosen as the solid-phase metal filler because it is a relatively inexpensive metal with a high thermal conductivity and good wettability for EGaIn [30]. First, the DEP-C-induced alignment of the metal fillers for the case of Cu, EGaIn, and the mixture of Cu and EGaIn (EGaIn-Cu) is compared. Next, the effects of the metal filler content on the thermal conductivity of the composite are evaluated with and without the DEP-C process. Finally, the performance of the pad-type EGaIn-Cu/PDMS TIM is evaluated by sandwiching it between a light-emitting diode (LED), as a heat source, and a heat sink and observing the temperature change over time.

## 2. Materials and Methods

### 2.1. Materials

EGaIn (Ga 75.5%/In 24.5%, ≥99.99% trace metals basis) and toluene (≥99.5%, extra pure) were purchased from Sigma-Aldrich, St. Louis, MO, USA. Copper (Cu) powder (spherical, 1 μm, ≥99.9% metals basis) was purchased from Avention, Siheung-si, Gyeonggi-do, Republic of Korea. PDMS (Sylgard 184) was purchased from Dow Corning Co., Ltd., Midland, MI, USA. Glass wafers with a chromium/gold (Cr/Au) coating (diameter of 4”) were received from AMED, Seoul, Republic of Korea.

### 2.2. Preparation of the Metal Filler/PDMS Compounds

First, 0.054–2.4 g of bulk EGaIn was placed in a vial filled with 1–2 mL of toluene and tip-sonicated for 12 min to disperse the EGaIn microparticles in the toluene. Around 0.05–0.6 g of Cu powder was then added to the dispersion of EGaIn particles for the biphasic fillers and then tip-sonicated again for 5 min to ensure that the EGaIn and Cu particles were fully mixed. The amount of EGaIn and Cu in the dispersion was adjusted to control the total amounts of the two metal fillers in the composites. After mixing, an appropriate amount of PDMS prepolymer was added to the dispersion and then heated to 120 °C on a hot plate for 2 h to evaporate the toluene. During the heating process, the dispersion was stirred at 500 rpm for 2 h using a magnetic bar. The uncured EGaIn-Cu/PDMS compound was cooled to room temperature, and the PDMS curing agent was added at a weight ratio of 1:10 to the previously added prepolymer. The uncured compound was stirred at 500 rpm for 10 min and then placed under vacuum for 40 min to remove residual toluene and entrapped air bubbles.

### 2.3. Observation of the Metal Filler/PDMS Compound under DEP-C

After the uncured EGaIn-Cu/PDMS compound was prepared, the behavior of metal fillers under DEP-C was observed. The EGaIn-Cu/PDMS compound was gently poured between two electrodes for DEP-C, which were made of a dual coating of Cr/Au with a thickness of 50 nm and 150 nm, respectively, on a glass substrate. The gap between the two electrodes was 500 μm. After the poured compound was sealed with a commercial incubation chamber (13 mm diameter × 0.2 mm depth, 22 mm × 25 mm OD, PC20) (Grace Bio-Labs, Inc., Bend, OR, USA), an alternating current (AC) with different frequencies was applied for 10 min. The dielectrophoretic behavior of the metal fillers under the electric field were observed using an optical microscope from the top view. The threshold function of ImageJ was used to obtain the binary images for the two-dimensional fast Fourier transform (2D FFT) method to remove the background blur from shadows and dust.

### 2.4. Fabrication of EGaIn-Cu/PDMS TIMs with Vertically Aligned Internal Fillers

To fabricate pad-type TIMs, a PDMS frame was prepared as a mold to hold the EGaIn-Cu/PDMS compound until it was cured. The prepared PDMS frame, with an inner space of 4 cm × 4 cm and a thickness of 500 μm, was placed on a Au-coated glass wafer, with a dimension of 6 cm × 6 cm. The EGaIn-Cu/PDMS compound was then poured into the PDMS frame and placed under vacuum for 20 min to remove air bubbles generated at the interface of the compound and the frame. After degassing was complete, another Au-coated glass wafer was gently placed on top of the PDMS frame filled with the EGaIn-Cu/PDMS compound and secured with clips to prevent leakage. These two Au-coated wafers at the top and bottom of the compound acted as electrodes during the DEP-C process. The compound, which was sandwiched between the electrodes, was then heated to 120 °C for 2 h to fully cure. At the same time, an AC of 140 Vpp at a frequency of 100 kHz was applied to the electrodes for the first 30 min of the curing process to induce the DEP-C of the metal fillers. After full curing, the TIM samples were carefully removed from the mold and cut to a proper size and stored.

### 2.5. Characterization

The morphological and microstructural properties of the compounds and TIMs were characterized using an optical microscope (Nikon, Tokyo, Japan, SMZ745T) and a high-resolution field-emission scanning electron microscope (FE-SEM) (Hitachi, Tokyo, Japan, SU8010). Optical image processing was conducted using the ImageJ software (v1.53e) to analyze the alignment of the metal particles. The size of the EGaIn and Cu particles in toluene was measured using a laser diffraction particle size analyzer (Beckman Coulter, Brea, CA, USA, LS 13 320). The selected samples were frozen using liquid nitrogen and shattered to pieces to observe the cross-section of the EGaIn-Cu/PDMS TIMs via SEM. The elemental composition of the EGaIn-Cu/PDMS TIMs was analyzed using energy-dispersive spectroscopy (EDS) (Hitachi, SU8010). The thermal conductivity of the TIMs was analyzed at 25 °C under atmospheric pressure in a nitrogen environment, using laser flash analysis (LFA) (Netzsch, Selb, Germany, LFA 467). The mechanical properties of the TIMs were measured using a universal testing machine (Instron, Norwood, MA, USA, E3000LT).

### 2.6. Thermal Performance Test of the TIMs

To test the thermal performance of the TIMs, they were sandwiched between the LED chip (10 W, 5 volts, 900 lumens, Shenzhen Chanzon Technology Co., Ltd., Shenzhen, China) and the heat sink (aluminum, 45 mm × 60 mm × 21 mm, Neato Trading, Shenzhen, China). The LED chip stayed on for 60 s after being turned on and was then turned off. The surface temperature of the LED chip was measured every 10 s using a thermal camera (Teledyne FLIR, Wilsonvile, OR, USA, FLIR ONE Gen 3). 

## 3. Results and Discussion

### 3.1. DEP-C-Induced Alignment of Biphasic Metal Particles

**Figure 2:**
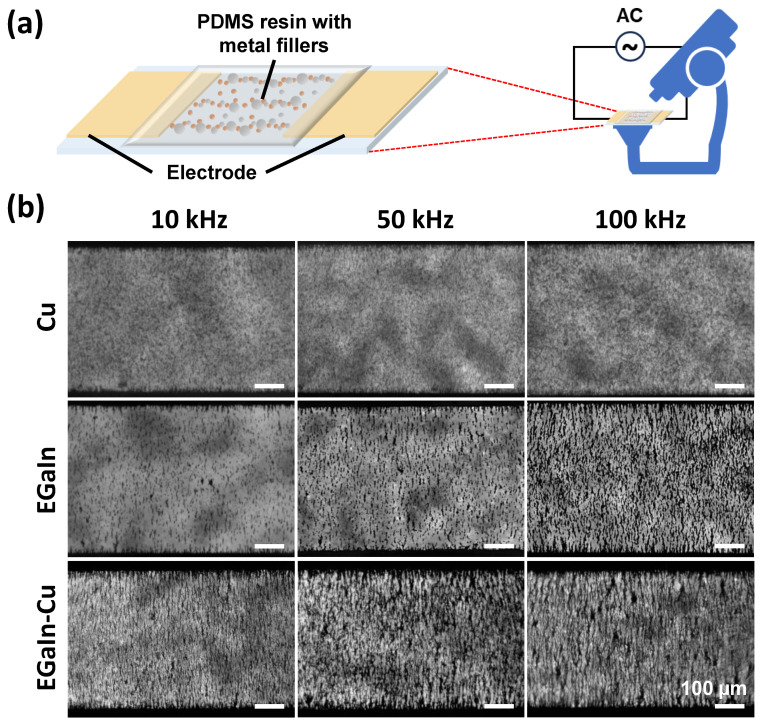
(**a**) Schematic of experimental observation of the EGaIn and Cu metal particles in the PDMS prepolymer under an AC electric field. (**b**) Optical microscope images showing the alignment of the metal particles in the PDMS resin. The AC electric field was applied at frequencies of 10, 50, and 100 kHz. The total amount of metal fillers in the PDMS prepolymer was 10 wt.% for all three samples. The ratio of EGaIn and Cu in the EGaIn-Cu sample was 1:1.

First, it was observed how well the metal particles were aligned under the AC electric field through the DEP-C mechanism in the PDMS prepolymer matrix. The degrees of alignment of only the Cu particles, only the EGaIn particles, and the EGaIn and Cu particle mixture were compared. To facilitate observation under the microscope, the uncured metal filler/PDMS compounds were placed between the two electrodes on a glass substrate, as shown in Figure 2a. The amplitude of the AC electric field was set to 140 Vpp, and its frequency was adjusted to 10, 50, or 100 kHz. The optical microscope images in Figure 2b show the degree of alignment of the EGaIn and Cu particles in the compounds after AC was applied for 10 min. No noticeable change was observed in the degree of alignment at all frequencies when only Cu particles were used as the filler material. However, the linear alignment of the particles appeared at a frequency of 50 kHz and was enhanced at 100 kHz when EGaIn particles were used as the filler material. For the EGaIn-Cu/PDMS compound, the alignment of particles was observed at all frequencies, and the linearity of the alignment was enhanced more as the frequency of the electric field increased. The EGaIn-Cu/PDMS compound showed the highest connectivity and linearity in the aligned particles, even at the lowest frequency, compared to the other compounds with only one type of metal filler, i.e., the Cu/PDMS and EGaIn/PDMS compounds.

**Figure 3:**
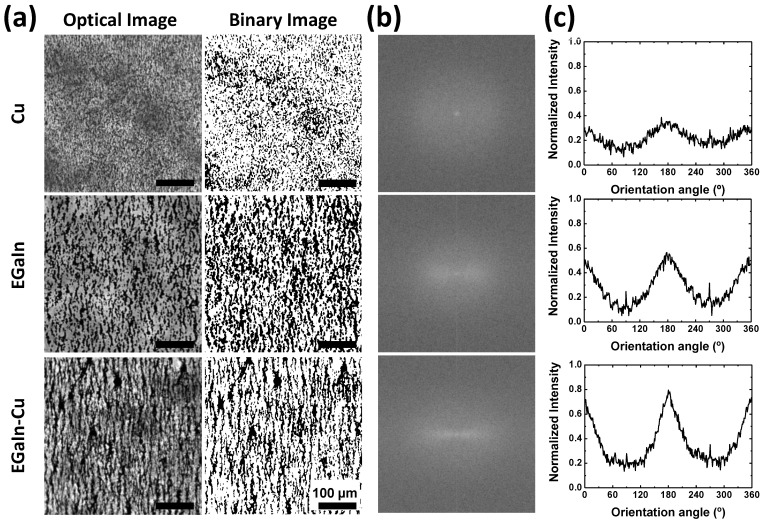
Quantitative evaluation of the electric field-induced metal particle alignment using the 2D FFT method. (**a**) Optical microscope images and binary images of only Cu particles, EGaIn particles, and EGaIn-Cu particle mixture in the PDMS prepolymer under an AC electric field (140 Vpp, 100 kHz). (**b**) 2D FFT outputs produced by ImageJ. The solid lines indicate the baseline where the orientation angle is 0°. (**c**) Orientation distribution plots from the radial sums of the FFT.

A 2D FFT method was applied (Figure 3) to quantitatively evaluate the degree of alignment of the metal particles in Figure 2 [32]. The first column of Figure 3a shows the optical microscope images and binary images of the Cu/PDMS, EGaIn/PDMS, and EGaIn-Cu/PDMS compounds under an AC field of 140 Vpp at 100 kHz. Figure 3b compares the resulting 2D FFT outputs from the binary images. Figure 3c plots the orientation distributions by conducting a radial summation of the pixel intensities of the FFT outputs in Figure 3b. The orientation angles are the angle counterclockwise from the horizontal, indicated by the solid lines. The peak intensity at the 180° angle in the orientation distribution plots indicates the degree of alignment of the metal particles along the electric field. The EGaIn-Cu mixture exhibits the highest intensity of the peak at the 180° angle. These results quantitatively support that the highest degree of alignment is induced by the AC electric field when both Cu and EGaIn particles are used as the filler materials in the PDMS matrix.

The DEP-C force depends on the size of particles, the permittivity of the particles and the medium, and the applied voltage. Assuming that the particles are homogeneous spheres, the force can be expressed in the following equation:

*F*(max) = −*Cε*_1_π*R*^2^*K*^2^*E*^2^, (1)

where *C* is the coefficient ranging from 3 to >1000, depending on the distance between the particles and the length of the particle chain; *ε*_1_ is the dielectric permittivity of the media; *R* is the particle radius; *K* is the Clausius–Mossotti factor; and *E* is electric field intensity [33].

**Figure 4:**
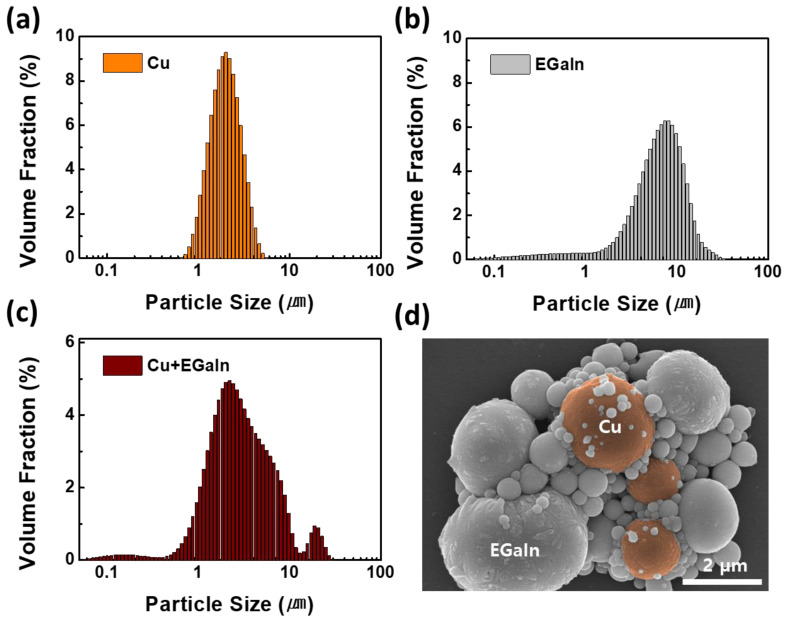
(**a**–**c**) Size distribution histograms of metal particles: (**a**) Cu particles; (**b**) EGaIn particles; (**c**) Cu and EGaIn particle mixture. (**d**) SEM image of the biphasic metal particle cluster, where the fused Cu and EGaIn particles are observed.

In the equation, the Clausius–Mossotti complex function, *K*, is the factor that accounts for the permittivity of materials. This function is not much different for all the compounds due to the infinite permittivity of the metal fillers, i.e., Cu and EGaIn. Thus, the size of the metal fillers in the matrix is the most critical factor in determining the difference in the DEP-C force. The size of the metal fillers is compared to investigate the reason why the EGaIn-Cu mixture shows the highest degree of alignment by DEP-C. Figure 4 represents the size distribution of the dispersed particles, Cu only, EGaIn only, and the EGaIn-Cu mixture. When only Cu particles are dispersed, most particle sizes are concentrated in a narrow range between 1 and 5 μm. When only EGaIn particles are prepared and dispersed, the particle size distribution range is wider, and most EGaIn particle sizes are around 10 μm, which is larger than the Cu particles. When EGaIn particles are prepared and dispersed with Cu particles, the broad distribution of EGaIn particles and the narrow distribution of Cu particles appear to overlap below 10 µm. Interestingly, a new peak appears in the range of 10–30 µm. As shown in the SEM images in Figure 4d and Figure S1, the Cu and EGaIn particles contact and fuse with each other, generating biphasic metal clusters. Larger biphasic metal clusters of 10–30 µm in size are observed in lower-magnification SEM images, as shown in Figure S2.

The biphasic metal fillers are likely formed due to the wetting property of EGaIn to Cu. When ultra-sonication is applied to the EGaIn-Cu mixture, the Cu and EGaIn particles not only coexist but fuse and form larger biphasic metal clusters. The binary phase diagram of Cu and Ga also supports that the two metals form alloys favorably at room temperature [34]. This easy alloy formation property promotes fusion between the Cu and EGaIn particles. The formation of the biphasic metal clusters effectively increases the particle size, enhancing the alignment driven by the DEP-C mechanism [35]. Moreover, the EGaIn particles between the Cu particles act as a bridging agent and improve the connectivity of fillers.

### 3.2. TIM Based on EGaIn-Cu/PDMS Compound 

**Figure 5:**
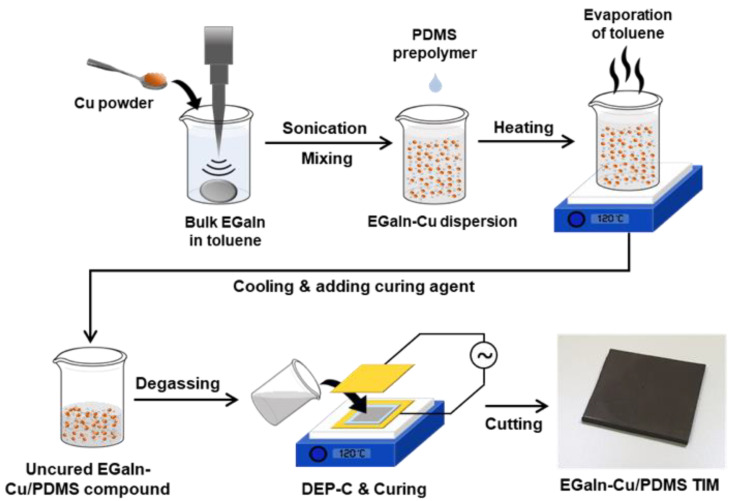
Schematic illustration of the fabrication process of pad-type EGaIn-Cu/PDMS TIM. Dimensions: 12 mm × 12 mm; thickness: 0.5 mm.

**Figure 6:**
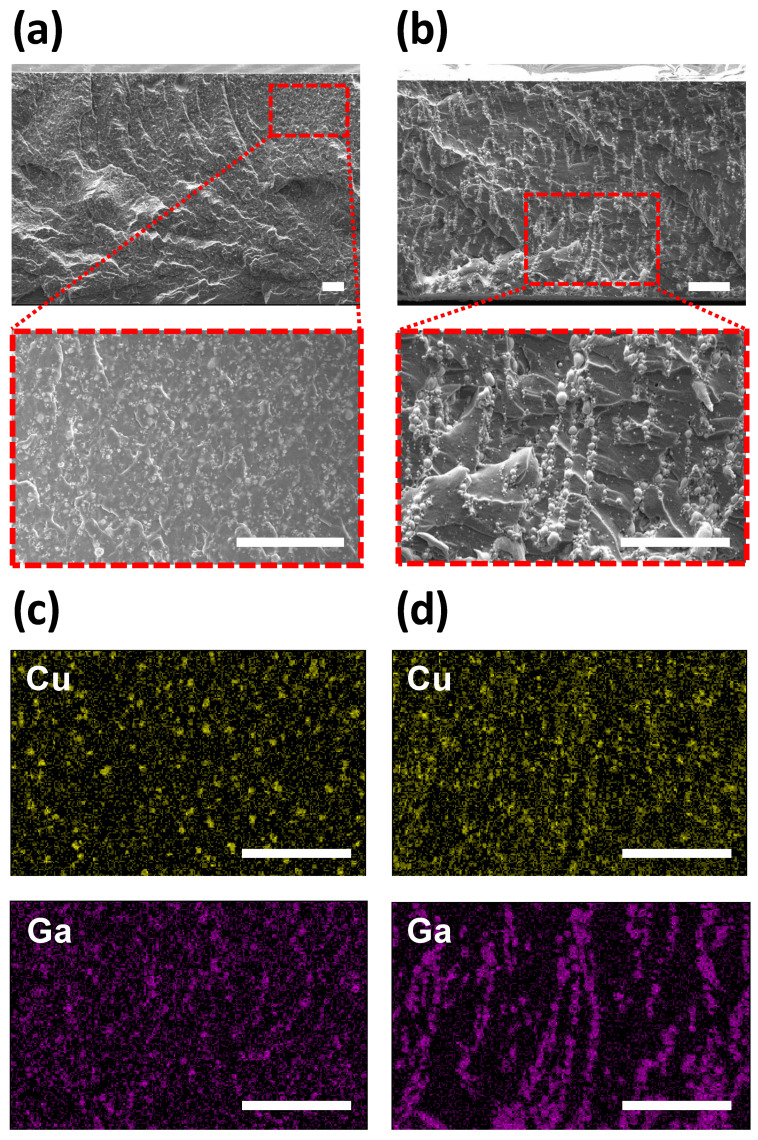
Cross-sectional SEM images and EDS analysis of the 48 wt.% EGaIn-Cu/PDMS TIM (**a**,**c**) without and (**b**,**d**) with the DEP-C process. Scale bar is 100 μm.

After confirming the alignment of metal particles by the DEP-C process, the pad-type TIMs are fabricated based on the EGaIn-Cu/PDMS compound. Figure 5 illustrates the fabrication process. In contrast to the experimental setup in Figure 2, the electrodes for DEP-C are placed at the top and bottom of the TIM to induce the vertical alignment of the metal particles. SEM and EDS were used to observe the cross-section of the EGaIn-Cu/PDMS TIM (Figure 6) to investigate the DEP-C-induced alignment of the metal fillers. The degree of alignment of the particles in the TIMs is compared with and without the DEP-C process. When the DEP-C process is not applied, the filler particles are just evenly dispersed in the composite without any noticeable alignment or chaining behavior. In contrast, when the DEP-C process is applied, the metal particles are well aligned, chaining vertically along the electric field direction. The EDS results in Figure 6c,d compare the elemental mapping results of Cu and Ga from EGaIn in the cured PDMS matrix with and without the DEP-C process. This result indicates that the DEP-C process linearly aligns the biphasic metal particles, which maintains the alignment in the cured TIM composites. It appears that EGaIn particles are relatively more responsive to electric fields, align better, and thus connect Cu particles.

### 3.3. Thermal and Mechanical Properties of EGaIn-Cu/PDMS TIMs

**Figure 7:**
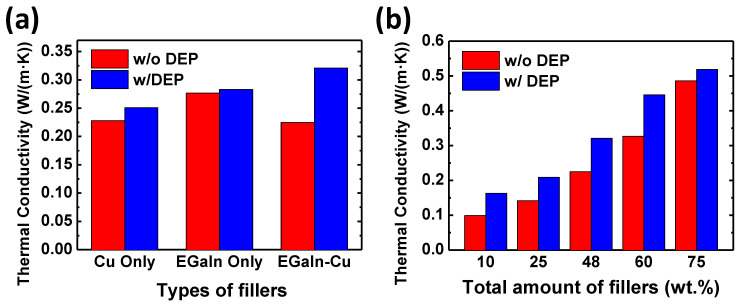
Effect of DEP-C-induced filler alignment on the thermal conductivities of TIM samples with (**a**) different fillers and (**b**) different total filler contents. The total filler content in (**a**) is 48 wt.%. The weight ratio of EGaIn and Cu is 4:1.

To ensure that the enhanced filler alignment in the EGaIn-Cu/PDMS effectively improves the thermal performance of the TIMs, Figure 7a compares the thermal conductivities of TIMs with different fillers with and without the DEP-C process. When the DEP-C process is not applied, the TIM with only the EGaIn particles exhibits higher thermal conductivity compared to those with only the Cu and biphasic particles. The EGaIn-only sample shows the highest thermal conductivity, probably due to the lower density of EGaIn than Cu (8.96 g/cm^3^ and 6.25 g/cm^3^ for Cu and EGaIn, respectively). That is, at the same weight, EGaIn has a larger volume compared to the Cu filler, resulting in higher thermal conductivity in the TIM composite. It should be noted that the filler volume is one of the most important factors in improving the thermal conductivity of the TIM [36]. When the DEP-C process is applied, the thermal conductivities of all TIM samples with different types of metal fillers increase. Particularly, the TIM with the biphasic metal fillers shows the most significant improvement of 42.6%, resulting in the highest value. This result is consistent with the results in Figure 2, Figure 3 and Figure 4, which display the enhanced DEP-C behavior of the Cu and EGaIn biphasic metal fillers. The filler alignment by DEP-C and better connectivity between the fused fillers help to improve the thermal conductivity of the TIM composite. Thus, utilizing biphasic metal particles with the DEP-C process can be very effective in improving the thermal conductivity of TIMs.

The effect of the total filler amounts on the thermal conductivity of the EGaIn-Cu/PDMS TIM was investigated. The filler amount varied from 10 to 75 wt.%. As the filler amount increases, the thermal conductivity also increases, regardless of whether the DEP-C process is applied or not. At all filler amounts, the thermal conductivity is improved when the DEP-C process is applied. When the filler amount is as high as 75 wt.%, the increase in thermal conductivity due to the DEP-C process is not significant compared to the TIMs with a lower filler amount. This is probably because the fillers are more prone to naturally connect with each other due to the high density at a high filler content, even without the DEP-C process. As a result, the effect of the DEP-C process on the thermal conductivity of TIMs is reduced.

**Figure 8:**
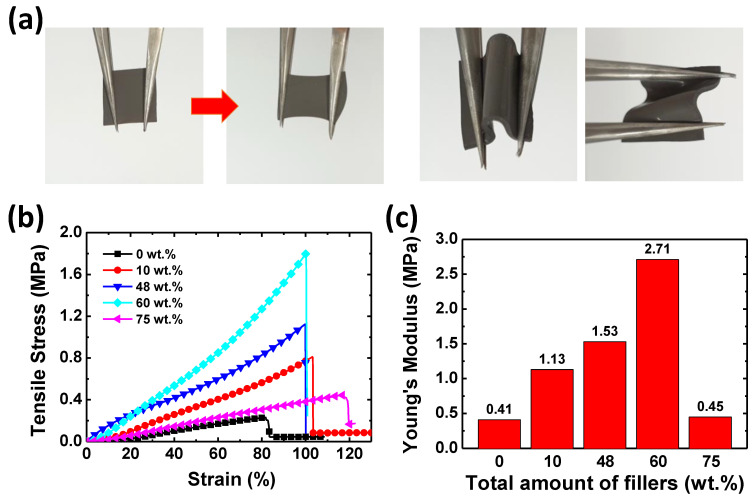
(**a**) Photographs of the pad-type EGaIn-Cu/PDMS TIM prototypes under mechanical deformation. The filler content is 48 wt.%. The size of the TIM sample is 12 mm × 12 mm, and the thickness is 0.5 mm; (**b**) strain–stress curve and (**c**) Young’s modulus of EGaIn-Cu/PDMS TIMs with different filler contents.

Pad-type TIMs should be soft and deformable enough to tightly fill the gap between the heat source and the heat sink and enable efficient heat dissipation. Figure 8a shows the resulting EGaIn-Cu/PMDS TIMs with a 48 wt.% filler content, which is highly soft, flexible, and stretchable. The TIM samples remained soft up to a 70 wt.% filler content. Uniaxial tensile strength tests were conducted to quantitatively examine how the filler amount influences the mechanical properties of the EGaIn-Cu/PDMS TIMs. Figure 8b shows tensile stress as a function of strain applied to the EGaIn-Cu/PDMS TIMs, with various filler contents ranging from 0 to 75 wt.%. The elongation at break increases as the metal fillers are introduced in the TIMs; no distinct trend is observed depending on the filler amount. Figure 8c compares the resulting elastic modulus values of the TIMs depending on the filler content. The modulus increases with the filler amount up to 2.71 MPa at 60 wt.%, which is approximately seven times higher than that of the pure PDMS (0.41 MPa). This result can be mainly attributed to the Cu filler, which exhibits a higher elastic modulus than the PDMS matrix. However, the elastic modulus drastically decreases to 0.45 MPa at 75 wt.%. Probably, the very high filler content degrades the structural integrity of the TIM, resulting in a decrease in the elastic modulus.

### 3.4. Thermal Performance of EGaIn-Cu/PDMS TIMs

**Figure 9:**
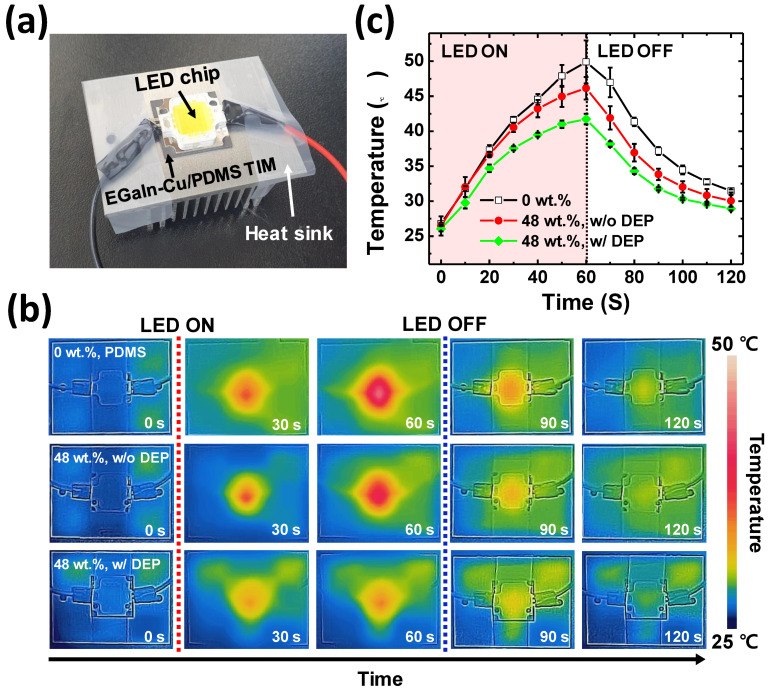
Heat dissipation performance of EGaIn-Cu/PDMS TIMs. (**a**) Photograph of the experimental setup; (**b**) time-dependent thermal images of the LED chip on pure PDMS TIM (**top**), EGaIn-Cu/PDMS TIM without the DEP-C process (**middle**), and EGaIn-Cu/PDMS TIM with the DEP-C process (**bottom**); (**c**) temperature change of the LED chip as a function of time.

As shown in Figure 9a, the TIM is inserted between the heat sink and the heat source (i.e., LED chip) to demonstrate the heat dissipation performance of the EGaIn-Cu/PDMS TIMs. The temperature change at the top surface of the LED is compared for three different TIM samples with the same size and thickness, namely the pristine PDMS TIM, the EGaIn-Cu/PDMS TIM without the DEP-C process, and the EGaIn-Cu/PDMS TIM with the DEP-C process. Figure 9b describes the result as time-dependent thermal images. The LED stayed on for 60 s before turning off. The LED chip generated heat once it was powered on, causing the temperature of the top surface to rise rapidly. Furthermore, even after the power was turned off, the residual heat did not dissipate immediately but persisted for more than 60 s. The pristine PDMS TIM shows the highest temperature increase; the EGaIn-Cu/PDMS TIM without the DEP-C process demonstrates a slight suppression in the temperature rise; and the EGaIn-Cu/PDMS TIMs with the DEP-C process exhibits the most effective heat dissipation performance with the least temperature increase. The graphs in Figure 9c depict the temperature changes of the LED on the different types of TIMs. The pristine PDMS TIM without fillers demonstrates the sharpest temperature rise, reaching almost 50 °C (92% increase over the initial temperature). The EGaIn-Cu/PDMS TIMs without the DEP-C process show a decrease in temperature change with the maximum temperature of 46 °C at 60 s (77% increase). The EGaIn-Cu/PDMS TIM with the DEP-C process demonstrates the lowest temperature rise to a maximum of 42 °C (62% increase). This thermal performance difference confirms that the enhanced alignment of EGaIn-Cu biphasic metal fillers by the DEP-C process improves the through-plane thermal conductivity of the TIM composite.

## 4. Conclusions

This study employs the DEP-C process to improve the anisotropic thermal conductivity of TIMs with biphasic metal fillers. The two filler materials, such as Cu and EGaIn particles, are selected for their high thermal conductivity and their ability to form alloys. The alignment and chaining of particles in a PDMS matrix are examined under different electric field conditions. The findings reveal that the highest linear alignment rate was achieved when both Cu and EGaIn particles were used as the biphasic metal fillers in the PDMS matrix. The aligned metal fillers enhanced the anisotropic thermal conductivity of the TIM composites. The heat dissipation performance of the TIMs in the direction of the filler alignment was confirmed by observing the temperature change of the LED chip connected to the heat sink through the TIMs. 

While the current values obtained in this paper may not surpass those reported recently, our study focused on an interesting phenomenon of the “DEP-C-induced alignment of metal fillers with two different phases”, aiming for higher thermal conductivity with a lower filler content. Future work to maximize the thermal conducting performance of TIMs with a minimal filler content involves controlling biphasic metal fusion; adjusting material compositions; optimizing manufacturing processes, including dispersion; and applying high-thermal-conductivity matrix materials.

As EGaIn wets several solid metals, such as silver in addition to copper, it is possible to investigate the biphasic metal fillers of EGaIn with those metals and their effect on the thermal performance of TIMs. Oxides, such as alumina and silica particles, which are commonly used as fillers of TIMs, are also promising candidates for fillers with EGaIn particles. The native oxide on the EGaIn surface can help to connect the metal particles with the oxide particles. Moreover, it would be interesting to know and understand how the particles behave under the DEP-C process as the metal and oxide particles have different permittivity under the electric field. Depending on the types of filler, the parameters of the DEP-C process could be tuned to enhance the heat dissipation performance of TIMs.

## Data Availability

The data in this study are available on request from the corresponding authors.

## Supplementary Materials

The following supporting information can be downloaded at: https://www.mdpi.com/article/10.3390/polym15244653/s1, Figure S1: Biphasic metal particle cluster composed of fused Cu and EGaIn particles; Figure S2: SEM image of large biphasic metal particle cluster composed of Cu and EGaIn particles of 10–30 µm in size.
