# Supplementary material for: The Dielectrophoretic Alignment of Biphasic Metal Fillers for Thermal Interface Materials
Source: Polymers (Basel). 2023 Dec 8;15(24):4653. doi: 10.3390/polym15244653 (PMC10747968; doi:10.3390/polym15244653)
Supplement: Supplementary file 1 [file polymers-15-04653-s001.zip › polymers-2744344-supplementary.pdf]

Supplementary Information

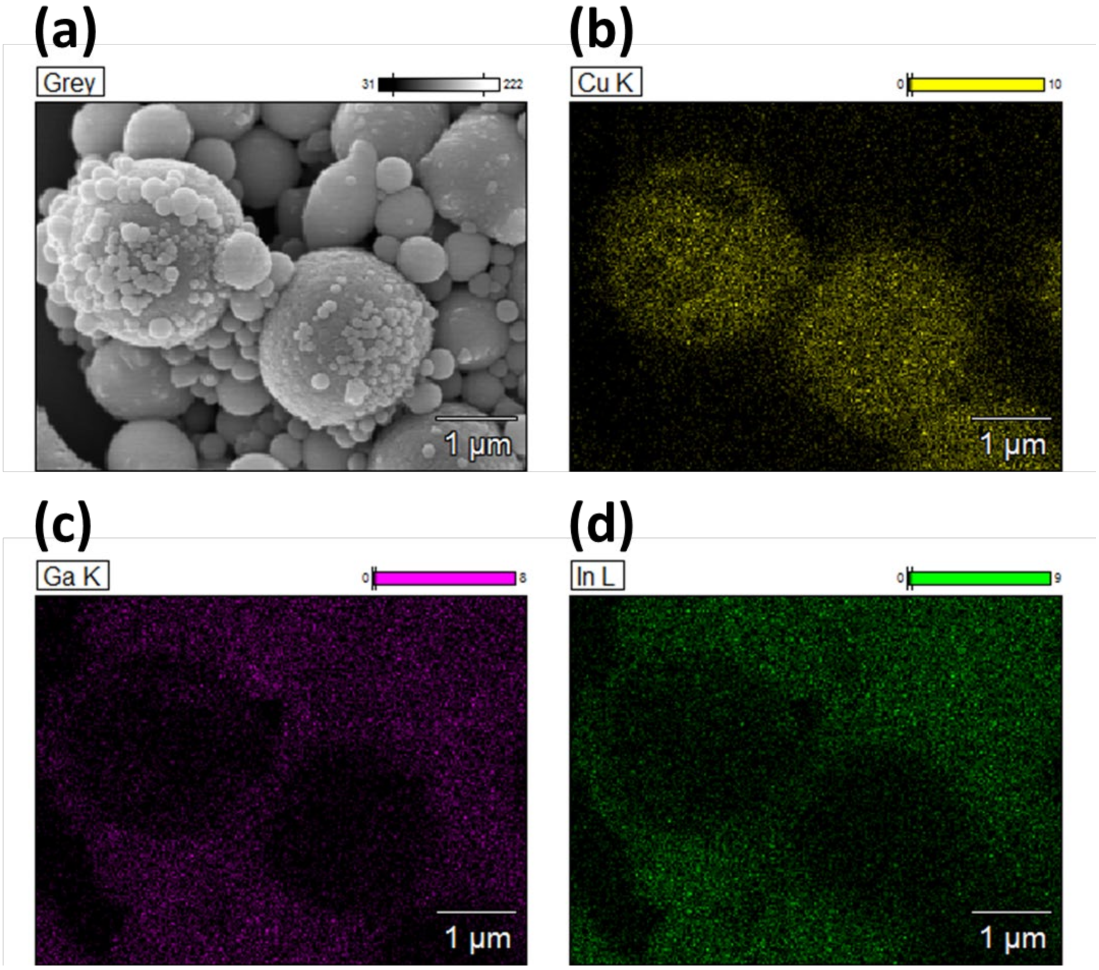

Supplementary Figure S1. Biphasic metal particle cluster composed of fused Cu and EGaIn particles; (a) SEM image and (b-d) EDS analysis.

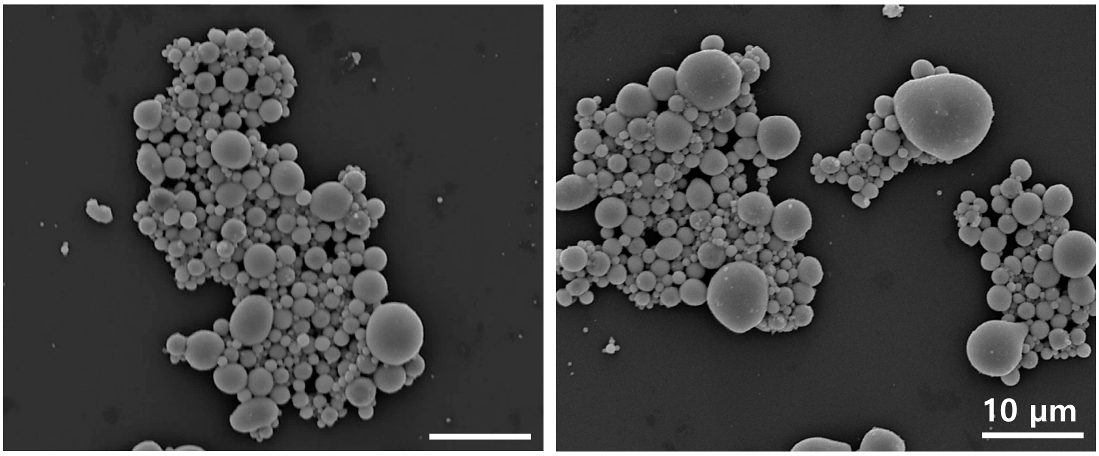

Supplementary Figure S2. SEM image of large biphasic metal particle cluster composed of Cu and EGaIn particles of 10-30 μm in size.
